# Diffusion controlled electrochemical analysis of MoS_2_ and MOF derived metal oxide–carbon hybrids for high performance supercapacitors

**DOI:** 10.1038/s41598-023-47730-4

**Published:** 2023-11-24

**Authors:** Vishal Shrivastav, Mansi, Prashant Dubey, Vaishali Shrivastav, Ashwinder Kaur, Marcin Hołdyński, Agnieszka Krawczyńska, Umesh K. Tiwari, Akash Deep, Wojciech Nogala, Shashank Sundriyal

**Affiliations:** 1 Institute of Physical Chemistry Polish Academy of Sciences, Kasprzaka 44/52, 01-224 Warsaw, Poland; 2 CSIR-Central Scientific Instruments Organisation, Sector 30-C, Chandigarh, 160030 India; 3 Advanced Carbon Products and Metrology Department, CSIR-National Physical Laboratory (CSIR-NPL), New Delhi, 110012 India; 4 Guru Nanak Dev University College, Chung, Punjab 143303 India; 5 Department of Physics, Punjabi University, Patiala, 147002 India; 6 Faculty of Materials Science and Engineering, Warsaw University of Technology, Wołoska 141 Str, 02-507 Warsaw, Poland; 7 Institute of Nano Science and Technology (INST), Sector-81, Mohali, Punjab, 140306 India; 8 Regional Center of Advanced Technologies and Materials, The Czech Advanced Technology and Research Institute (CATRIN), Palacký University Olomouc, Šlechtitelů 27, 779 00 Olomouc, Czech Republic

**Keywords:** Coordination chemistry, Electrochemistry, Energy, Materials for energy and catalysis, Nanoscale materials

## Abstract

In the context of emerging electric devices, the demand for advanced energy storage materials has intensified. These materials must encompass both surface and diffusion-driven charge storage mechanisms. While diffusion-driven reactions offer high capacitance by utilizing the bulk of the material, their effectiveness diminishes at higher discharge rates. Conversely, surface-controlled reactions provide rapid charge/discharge rates and high power density. To strike a balance between these attributes, we devised a tri-composite material, TiO_2_/Carbon/MoS_2_ (T10/MoS_2_). This innovative design features a highly porous carbon core for efficient diffusion and redox-active MoS_2_ nanosheets on the surface. Leveraging these characteristics, the T10/MoS_2_ composite exhibited impressive specific capacitance (436 F/g at 5 mV/s), with a significant contribution from the diffusion-controlled process (82%). Furthermore, our symmetrical device achieved a notable energy density of ~ 50 Wh/kg at a power density of 1.3 kW/kg. This concept holds promise for extending the approach to other Metal–Organic Framework (MOF) structures, enabling enhanced diffusion-controlled processes in energy storage applications.

## Introduction

In the landscape of contemporary energy storage devices, capacitors and batteries emerge as two pivotal players poised to meet the burgeoning demand^1^. Batteries boast remarkable energy density but falter when it comes to high-power output, often succumbing to safety concerns such as short-circuiting and metal dendrite formation during rapid charge/discharge cycles^2,3^. Electrostatic capacitors have historically been employed to bolster high-power delivery in various applications^4,5^, albeit marred by their relatively low energy density. In recent decades, electrochemical capacitors, with energy densities ranging from 0.01 to 10 Wh/kg, have bridged the gap between power and energy storage, surpassing the capabilities of their electrostatic counterparts^6^. The trajectory of research now leans toward hybrid supercapacitor designs, featuring one electrode with capacitor-like attributes and another with battery-like characteristics to augment energy density^7^. This paradigmatic shift empowers capacitor-type electrodes to deliver high power density, while battery-type counterparts offer elevated energy density. For example, Gao and his team harnessed Sri Lanka graphite ore as an electrode material, achieving an impressive energy density of 86 Wh/kg in organic electrolytes^8^. Similarly, Chen engineered a carbon framework with nitrogen doping for the anode and employed activated carbon for the cathode in a hybrid supercapacitor, yielding 172 Wh/kg of energy density^9^. However, these notable energy densities in tandem with organic electrolytes, while effective, necessitate stringent inert conditions and raise fire safety concerns in adverse scenarios. Moreover, the adoption of hybrid designs introduces complexity and increased costs, diverging from the simplicity offered by symmetrical supercapacitors, which are favored by industries. To tackle these challenges, researchers are now exploring hybrid materials endowed with conductivity, extensive surface area, and redox-active sites. The integration of metal oxides or conducting polymers into carbon-based materials has demonstrated promising potential in enhancing supercapacitor performance^10,11^.

To design an effective hybrid material for supercapacitors, it’s imperative to comprehend the underlying charge storage mechanisms. Supercapacitors primarily store charges through either the adsorption/desorption of electrolyte ions or redox activity, with the efficient utilization of the material being pivotal for enhancing energy density^12^. Given that supercapacitors operate at rapid charge/discharge rates, a significant portion of the material often remains underutilized due to diffusion limitations within the bulk. To address this challenge, creating a highly porous material becomes essential to facilitate ion access throughout the bulk. However, many redox-active materials possess high density but limited ion diffusion capability, while non-redox porous materials lack high energy density. Hence, a hybrid material with both a porous core structure and a densely packed redox-active surface is envisioned to provide high energy density through surface redox reactions and efficient ion diffusion within the bulk.

In the quest for a porous core structure, we employed the MIL-125 MOF structure to synthesize a TiO_2_/carbon composite. Titanium dioxide (TiO_2_) was chosen for its exceptional faradaic capacitance and robust physical and chemical stability^13,14^. However, its application in energy storage systems has been hindered by high internal resistance. Researchers have explored composites of TiO_2_ with conductive structures, such as activated carbon, carbon nanotubes, and reduced graphene oxide, to improve conductivity^15–18^. Although these composites have enhanced electrochemical performance, challenges persist due to weak van der Waals forces, poor TiO_2_/carbon interfaces, and nanoparticle aggregation. Metal–organic frameworks (MOFs), three-dimensional structures composed of metal centers and organic linkers, have emerged as a versatile platform^19^. While most MOFs are non-conductive, their pyrolysis yields nanoporous carbon with embedded metals that can form metal oxides^20,21^. This approach resulted in TiO_2_ nanoparticles distributed within the carbon framework, albeit with a limited oxide fraction. To enhance surface redox activity, MoS_2_ nanosheets were chosen for their high theoretical capacity of 1290 mAh/g^22–24^. Li et al. showed the efficacy of a core–shell nanocomposite for supercapacitor applications. When discharged at the current density of 2.55 mA/cm^2^, CeO_2_@MoS_2_ delivered 90 mF/cm^2^ of C_s_ which is much higher as compared to pure MoS_2_ nanosheets (40 mF/cm^2^)^25^. Similarly, Kim et al. utilized a CeO_2_/carbon hybrids having high porosity and redox activity for supercapacitors which delivered an improved C_s_ of 280 F/g^26^. These studies indicate that the use of porous carbon to improve the electrochemical performance of non-conducting redox active material, such as MoS_2_ and CeO_2_, is a promising strategy. Overall, the literature suggests that nanocomposites based on oxides, MoS_2_, and carbon exhibit superior capacitance compared to their individual counterparts^27^.

In this study, we synthesized a tri-composite material where TiO_2_ is integrated into the carbon framework and further decorated with MoS_2_ nanosheets to bolster surface redox activity and ion diffusion within the porous carbon structure during rapid charge/discharge cycles, ultimately yielding high energy density. The resulting TiO_2_/Carbon/MoS_2_ (T10/MoS_2_) exhibited a specific capacitance of 470 F/g with 70% capacitance retention after 4000 charge/discharge cycles in a three-electrode system using 6 M KOH. Remarkably, at very high scan rates, the material displayed significant current contributions (60%) from diffusion, surpassing TiO_2_/carbon (31%) and MoS_2_ (36%). Furthermore, an all-solid-state symmetrical supercapacitor was constructed using T10/MoS_2_ as an electrode, polyvinyl alcohol (PVA) as a gel electrolyte, and separator, achieving a capacitance of 192 F/g at a discharge rate of 0.5 A/g. Notably, the device delivered an energy density of 49.8 Wh/kg at a power density of 1.3 kW/kg and retained 16.7 Wh/kg of energy density even at higher discharge powers of up to 16.25 kW/kg. This innovative tri-composite material represents a promising advancement in supercapacitor technology, addressing the critical need for efficient energy storage solutions.

## Result and discussion

### Material characterization

**Figure 1:**
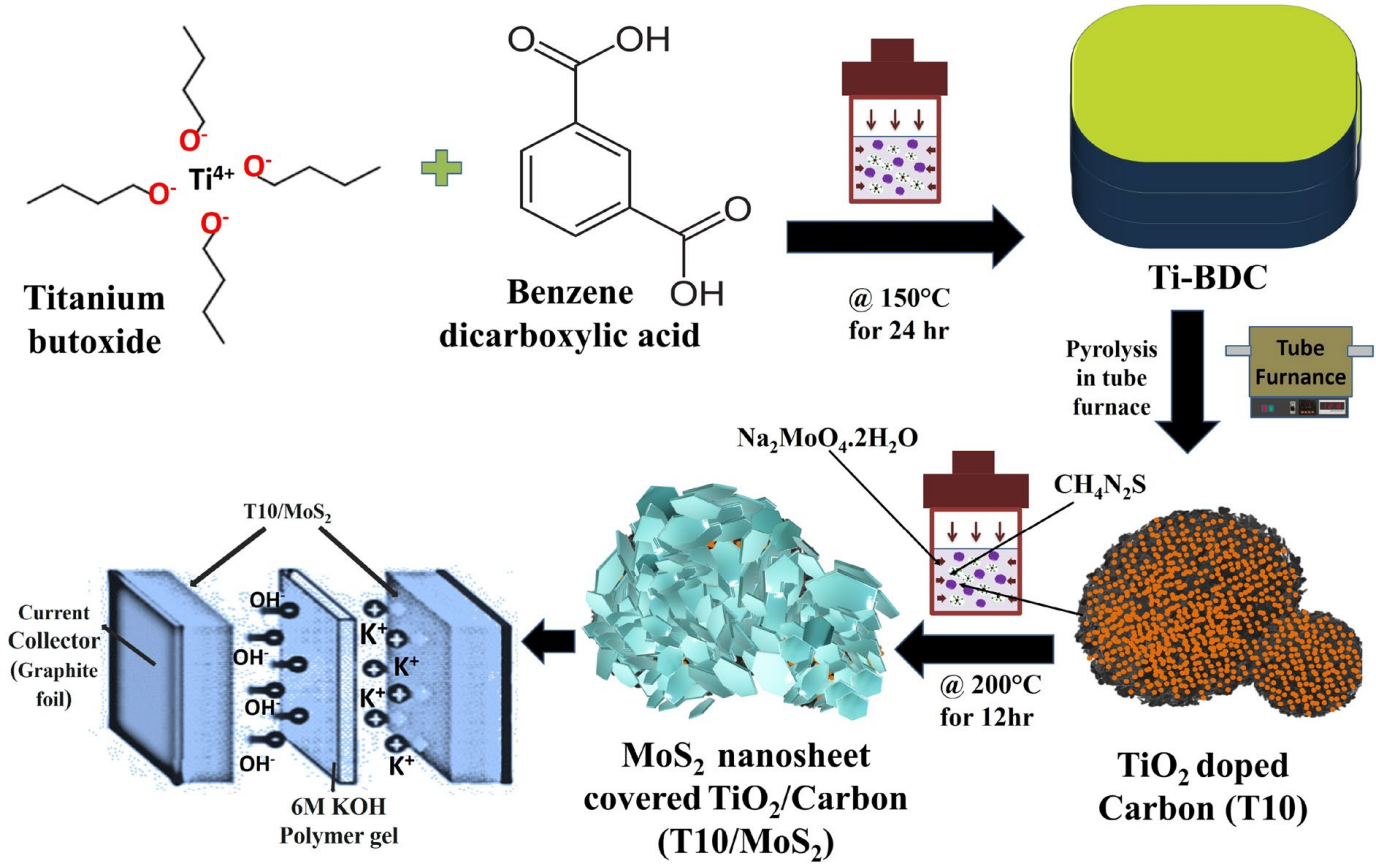
Schematic of the synthesis of MoS_2_ nanosheet covered TiO_2_/carbon and its application for energy storage devices.

The experimental procedure and conditions for material synthesis are detailed in the supplementary file (SI) in the S1 section. Figure 1 illustrates the synthesis process for T10/MoS_2_ material. A one-pot synthesis approach was employed to create Ti-MOF under solvothermal conditions, yielding a light-yellow powdered product. XRD characterization confirmed the characteristic peaks of Ti-MOF, as depicted in Figure S1 of the SI. Subsequently, Ti-MOF underwent pyrolysis in an argon (Ar) atmosphere at three distinct temperatures (600, 800, and 1000 °C), denoted as TN (where N represents 6, 8, and 10 corresponding to pyrolysis temperatures of 600, 800, and 1000 °C, respectively). MOFs inherently consist of uniformly distributed metal clusters and organic linkers with high crystallinity. Consequently, during pyrolysis, the organic components transformed into a nanoporous carbon structure with the uniform dispersion of nanoscale metal oxides. This strategic approach facilitated the production of a porous carbon structure infused with metal oxides.

Or porosity and surface area analysis, N_2_ adsorption–desorption studies were conducted on the TN samples (Figure S2). Among them, T10 exhibited the highest specific surface area (SSA) at 262 m^2^/g, surpassing T8 and T6, which recorded 186 m^2^/g and 50 m^2^/g, respectively. Analysis of the pore size distribution curve revealed that the T10 sample displayed a higher frequency within the mesoporous range (2–50 nm). Additionally, FTIR analysis of the T10 sample was conducted to identify surface groups capable of nucleating MoS_2_ nanosheets through Mo–O and Mo–OH bonding (Figure S3). The analysis indicated the presence of carboxylic groups and Ti–O groups in the 500–1000 cm^−1^ band, along with Ti–OH groups in the 1500–1750 cm^−1^ range. These groups can potentially initiate the growth of MoS_2_ on the T10 surface. Based on its high SSA and pore size distribution, T10 was selected as the substrate for MoS_2_ nanosheet growth.

**Figure 2:**
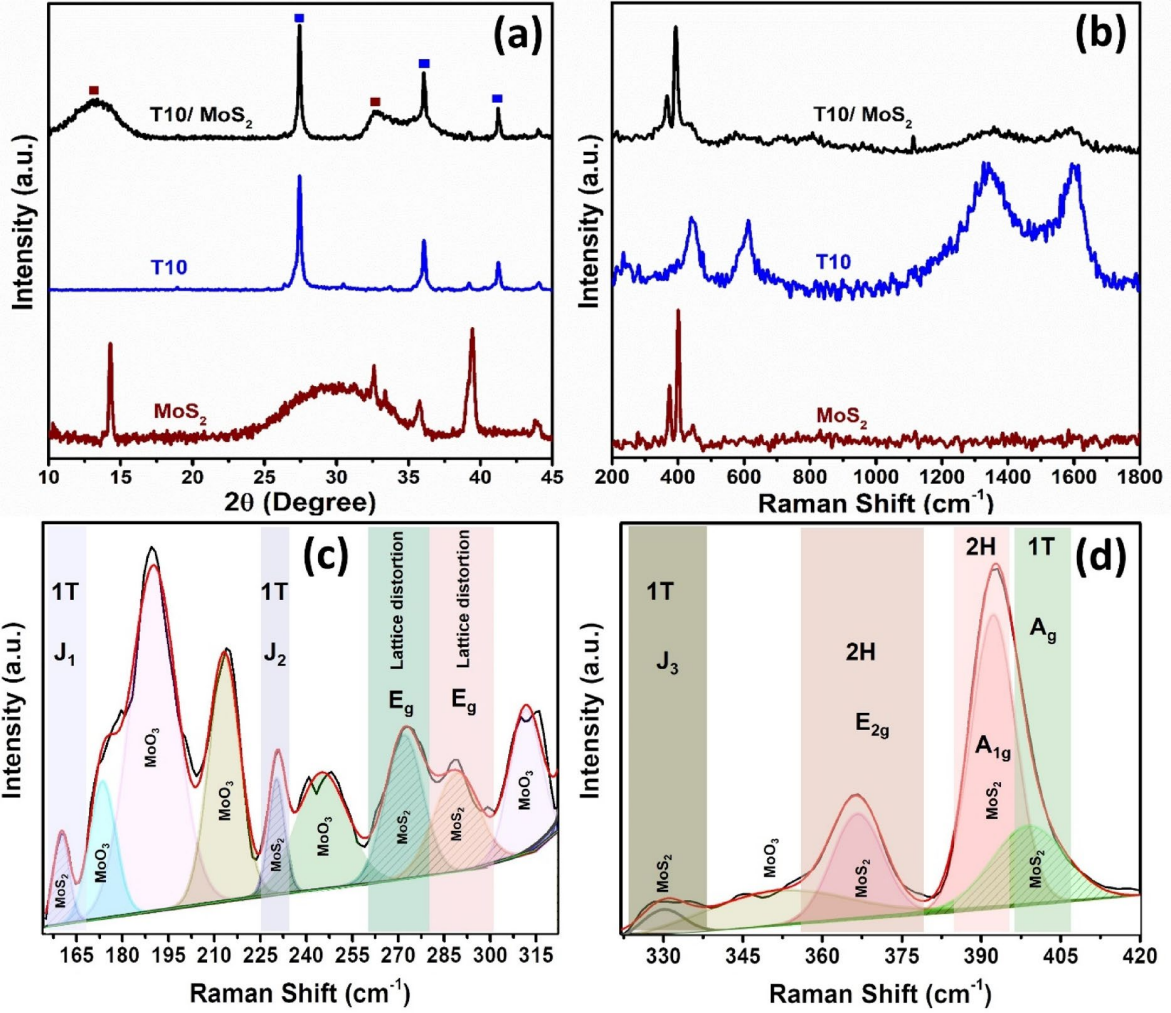
(**a**) Comparative XRD of T10, T10/MoS_2_, and MoS_2_, (**b**) comparative Raman spectra T10, T10/MoS_2_, and MoS_2_, (**c**) deconvoluted Raman spectra of T10/MoS_2_ from 155–315 cm^−1^, and (**d**) deconvoluted Raman spectra of T10/MoS_2_ from 320–420 cm^−1^.

The synthesis of MoS_2_ on the T10 structure (T10/MoS_2_) followed a one-pot method, initiated by the attachment of Mo to the negatively charged oxygen atoms in the carbon structure, serving as nucleation centers for MoS_2_ nanosheet growth^28^. The prepared sample has been characterized with the XRD to determine the composite formation and other crystallographic properties. The XRD of the T10/MoS_2_ showed the presence of rutile phase of TiO_2_ (JCPDS No. 21–1276) (shown in Fig. 2a)^29^. Notably, the peaks associated with TiO_2_ remained unchanged when comparing XRD patterns of T10 and T10/MoS_2_, indicating no structural alterations in TiO_2_ during the hydrothermal reaction. However, when examining MoS_2_ peaks, those in the composite were broadened and some were absent compared to bulk MoS_2_. This could be attributed to distortion in d-spacing and limited long-range order, likely caused by the growth of MoO_x_ on the T10 surface, with oxygen groups (C–O, Ti–OH) acting as nucleation centers ^30^. Further, the growth of MoS_2_ is initiated through MoO_x_ which produced distorted MoS_2_ having 1 T phase followed by 1H phase with the decrease of lattice distortion. Although, 1 T MoS_2_ is not as stable as 1H phase, however the presence of lattice distortion could favors octahedrally coordinated Mo atoms (1 T) instead of trigonal-prismatic structure (2H)^30,31^. For electrochemical activity, the distorted metallic 1 T-MoS_2_ shows higher activity relative to the 2H phase on both the edge sites and the basal plane^32^.

Therefore, to confirm the presence of 1 T-MoS_2_, 2H-MoS_2_, and MoO_x_, we have performed Raman spectroscopy for the samples (Fig. 2b)^33^. The comparative Raman spectra of MoS_2_, T10, and T10/MoS_2_ shows the presence of E_2g_ and A_1g_ mode of MoS_2_ at 360–420 cm^−1^ and defective (D) and G band of carbon at 1300–1600 cm^−1^. The low intensity of Ti–O and carbon when compared to MoS_2_ is due to the presence of bulk MoS_2_ at the surface. The bulk MoS_2_ at the surface thus give strong intensity peaks compared to carbon and TiO_2_ which are underneath the sample. The presence of 1 T MoS_2_ and MoO_x_ has been confirmed through the deconvolution of Raman peaks from 155–420 cm^−1^ (Fig. 2c,d). The deconvolution of T10/MoS_2_ sample for 155–315 cm^−1^ shift shows the presence of peaks at 160 cm^−1^ and 230 cm^−1^ which is assigned to the J_1_ and J_2_ mode of 1 T MoS_2_^34^. Further the presence of peaks at 270–290 cm^−1^ is the E_g_ mode which arises due to the distortion in 1 T MoS_2_^35^. The other Raman shift are the result of the vibrations modes of Mo-O^30^. The deconvolution of Raman spectra from 320–420 cm^−1^ reveals the presence of 1 T MoS_2_ and 2H MoS_2_ bands (Fig. 2d). The shift at 330 cm^−1^ is the J_3_ mode of 1 T MoS_2_, whereas the peaks at 366 cm^−1^ and 392 cm^−1^ is the E_2g_ and A_1g_ mode of 2H MoS_2_. The broadening and shortening the E_2g_ peak of 2H–MoS_2_ is also the sign of the presence of 1 T MoS_2_ phase. In addition to this, the softening of the 2H MoS_2_ shift is related to the distortion in the lattice and the presence of 1 T MoS_2_ phase. The deconvoluted peaks at 398 cm^-1^ is assigned to the Raman shift related to the Ag mode of 1 T MoS_2_ confirming the presence of MoO_x_, 1 T and 2H MoS_2_. The same time of growth MoS_2_ with 1 T-MoS_2_ at the interfaces of carbon derivative and 2H-MoS_2_ has also been observed in literature^36^. The XRD and Raman spectroscopy of the T10/MoS_2_ sample confirmed the successful growth of MoS_2_ nanosheets over the T10 samples.

**Figure 3:**
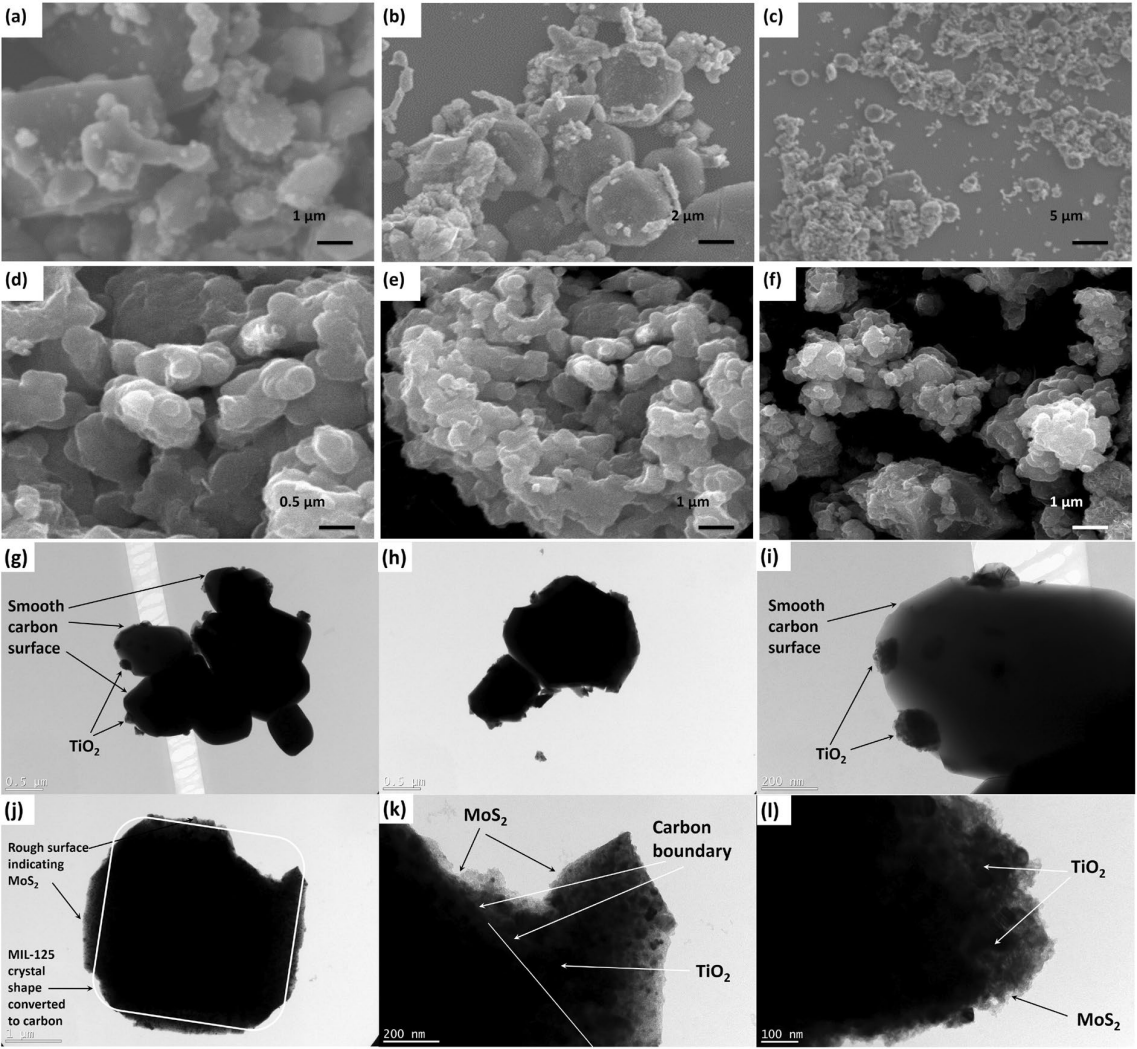
FESEM images of (**a**–**c**) T10 and (**d**–**f**) T10/MoS_2_; TEM images of (**g**–**i**) T10 and (**j**–**l**) T10/MoS_2_.

Field-emission scanning electron microscopy (FESEM) was employed to investigate the structural morphology of the T10 and T10/MoS_2_ samples (Fig. 3a,b,c,d,e,f). The FESEM images of the T10 sample revealed a spherical carbon structure with TiO_2_ nanoparticles uniformly dispersed throughout, confirming the distribution of TiO_2_ within the conductive carbon framework (Fig. 3a,b,c). To check the conductivity of the T10 sample, we performed the IV studies from which the sample showed 636 Ω of in plane resistance when tested on 0.5 cm of pallet of T10 material prepared with the hydraulic press (Figure S4). In contrast, the FESEM images of the T10/MoS_2_ sample exhibited carbon spheres enveloped by MoS_2_ nanosheets, providing visual evidence of the tri-composite formation (Fig. 3d,e,f). This tri-composite structure possesses the advantageous combination of surface redox activity and a porous, conductive core. Ideally suited for pseudocapacitance, this material features a porous core that facilitates rapid ion transport for efficient charge transfer, while the surface redox activity ensures swift electrochemical reactions. The presence of redox-active MoS_2_ on the surface and the conductive, porous T10 core synergistically contribute to high capacitance, offering an ideal distribution of electrode material at the nanoscale.

Further to clearly see the morphological characteristics of the material, we performed the TEM analysis given in Fig. 3g,h,i,j,k,l. The T10 sample showed in Fig. 3g,h,i showed the carbon structures with the protruding TiO_2_ nanostructures. The carbon surface is completely smooth and TiO_2_ is dispersed at the surface. Although the dispersion of TiO_2_ is not uniform but randomly distributed probably depend on the temperature gradient in the tube furnace and the impurities in the crystal which allowed the break of bonds from that place and protruding TiO_2_ out of the structure. The TEM image for T10/MoS_2_ is shown in Fig. 3j,k,l. The carbon which had a smooth surface in T10 had rough surface in T10/MoS_2_ indicating the growth of MoS_2_ over it. The structure of the MIL-125 crystal retained as carbon structure can be seen in the Fig. 3j. In the higher magnified image (Fig. 3k), the MoS_2_ can be seen at the top, TiO_2_ can be seen coming out and carbon boundary is recognizable. Further magnifying the sample (Fig. 3l), the carbon structure is completely covered with MoS_2_ nanosheet which confirm the successful formation of T10/MoS_2_ sample.

**Figure 4:**
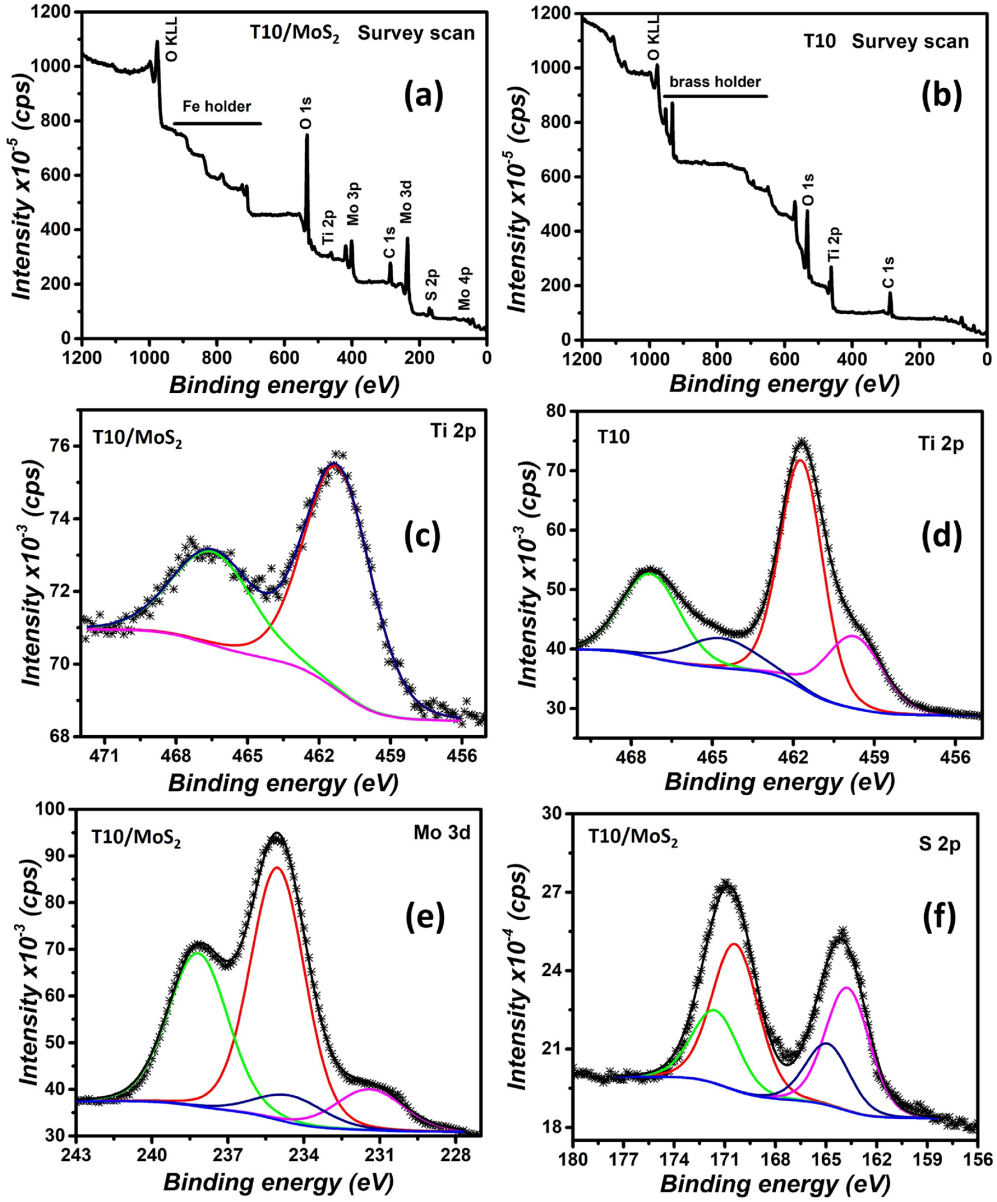
(**a**) T10/MoS_2_ survey scan, (**b**) T10 survey scan, (**c**) high resolution Ti 2p spectra for T10/MoS_2_, (**d**) high resolution Ti 2p spectra for T10/MoS_2_, (**e**) high resolution Mo 3d spectra for T10/MoS_2_, and (**f**) high resolution S 2p spectra for T10/MoS_2_.

Further, we also performed XPS analysis for T10 and T10/MoS_2_ to determine the presence of different elements and their chemical state. The survey scan of T10/MoS_2_ and T10 is given in Fig. 4a,b respectively. The survey scan of T10/MoS_2_ demonstrate the presence of C, Ti, O, S, and Mo which is due to the presence of carbon, TiO_2_, and MoS_2_. Further the sample T10 only contains C, Ti, and O correspond to carbon and TiO_2_. There are some peaks related to the sample holder which is marked in the Fig. 4a,b. The high-resolution spectra of Ti for T10/MoS_2_ and T10 is also plotted which is given in Fig. 4c,d respectively. The sample T10 contains the Ti 2p_3/2_ peak at 461.8 eV and Ti 2p_1/2_ at 467.3 eV, indicating the + 4 state of Ti correspond to TiO_2_^37^. However, there are weaker peaks also at 459.7 eV and 464.7 eV which indicated the + 3 oxidation state of Ti attributed to the presence of oxygen vacancy and incomplete oxidation of Ti in T10 sample^38^. However, in case of T10/MoS_2_ sample, there are no peaks correspond to + 3 oxidation state which indicates the full oxidation of Ti while doing hydrothermal synthesis step to grow MoS_2_. Further, to check the Mo bonding we analyzed the high resolution spectra of Mo as shown in Fig. 4e. Mo 3d spectra is convoluted into 3d_5/2_ and 3d_3/2_ peaks at 235 eV and 238.2 eV respectively attributed to the Mo-S bonding. There are two weaker bonds also at 231.3 eV and 234.7 eV which can be assigned to the Mo–O bonds^39^. The high resolution spectra of S 2p is also given in Fig. 4f which is deconvoluted into 2p_3/2_ and 2p_1/2_ at 163.7 eV and 164.9 eV respectively correspond to the Mo-S bonding energy^40^. The other weaker peak assigned to 2p_3/2_ at 170.4 eV can be assigned to the C–S–H bonds.

**Figure 5:**
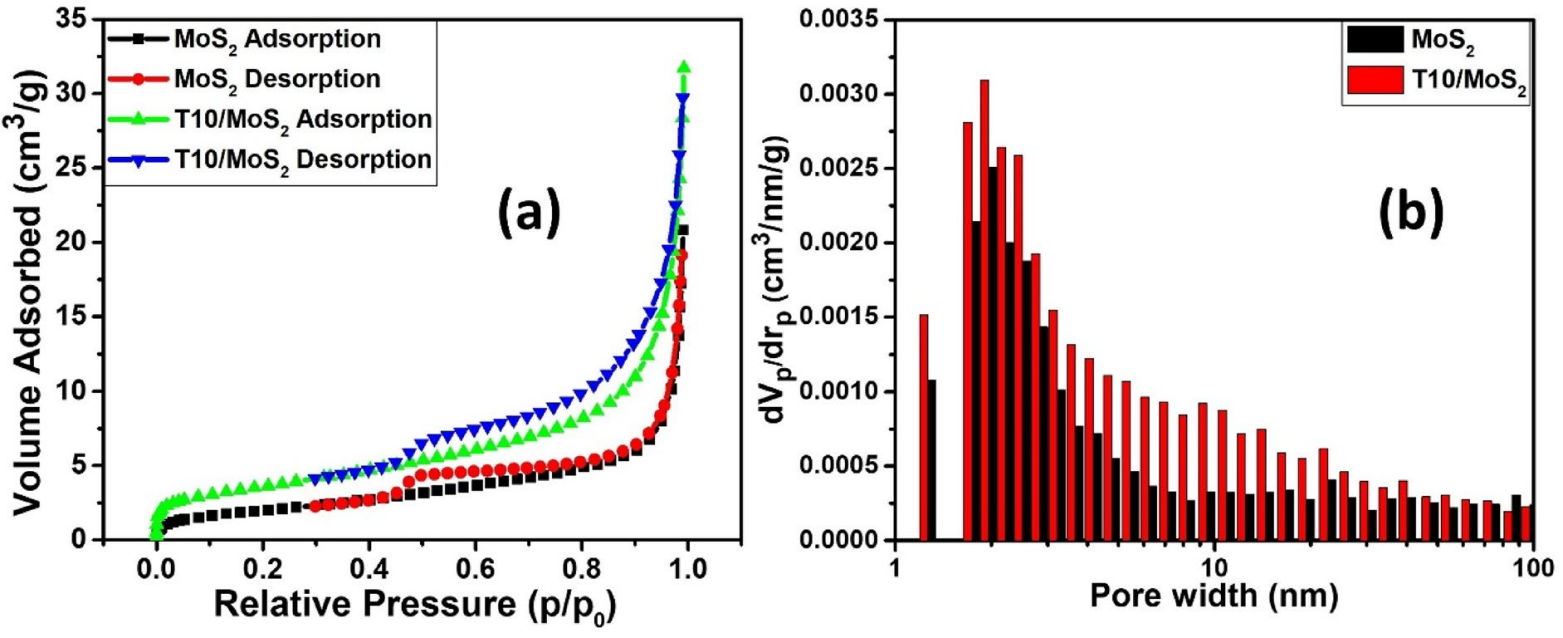
(**a**) N_2_ adsorption desorption isotherm and (**b**) Pore size distribution curve of T10 and T10/MoS_2_.

The nitrogen adsorption–desorption isotherms displayed in Fig. 5a offer valuable insights into the porous characteristics and surface properties of the materials under investigation: pure MoS_2_ and the composite T10/MoS_2_. The T10/MoS_2_ sample achieved the surface area of 32 m^2^/g whereas MoS_2_ exhibit the 7 m^2^/g of SSA. Further, the decrease in specific surface area from 262 m^2^/g in T10 to 32 m^2^/g in T10/MoS_2_ can be explained by the introduction of MoS_2_ nanosheets on the surface of T10 during the synthesis process. While it may appear that the porous structure of T10 is damaged in T10/MoS_2_, this reduction in specific surface area does not necessarily contradict the conclusion but instead points to a different aspect of the composite's electrochemical performance. The decrease in specific surface area can be attributed to the MoS_2_ nanosheets covering the surface of T10. The presence of MoS_2_ on the surface may partially block or fill some of the pores, reducing the overall specific surface area. However, this reduction in surface area is compensated by the addition of redox-active MoS_2_ nanosheets, which contribute to the overall capacitance through surface-controlled redox reactions. The higher SSA of T10/MoS_2_ compared to MoS_2_ can be attributed to the porous carbon core. The presence of porous core can benefit the fast transfer of ions in the bulk material due to presence of mesoporosity confirmed from the pore size distribution of T10/MoS_2_ Upon a close examination of the isotherm for pure MoS_2_, it becomes evident that there is an initial gradual increase in nitrogen uptake at low relative pressures (P/P_o_). This behavior is indicative of the presence of microscale pores within the pure MoS_2_ material. As the gas pressure increases, a greater quantity of nitrogen molecules adheres to the material's surface and permeates these microscale pores. It's noteworthy that as these micropores become saturated, the rate of nitrogen uptake progressively diminishes. On the other hand, the nitrogen adsorption isotherm of T10/MoS_2_ reveals a more complex pattern compared to pure MoS_2_. Most notably, the initial uptake of nitrogen is significantly higher when compared to MoS_2_, particularly at low pressures. This substantial adsorption at low pressures suggests the existence of an extensively porous structure within the composite material. This porous structure is characterized by a network of both microscale and mesoscale pores, contributing to the composite's considerably larger surface area, as discussed previously. As the relative pressure continues to rise, there is a remarkable surge in nitrogen adsorption by the composite. This behavior signifies the presence of additional sorption sites within the material and indicates capillary condensation occurring within the mesoscale pores. Capillary condensation is a phenomenon in which gas molecules are drawn into mesopores due to capillary forces arising from the restricted pore size, leading to a sudden and substantial increase in adsorption. The desorption phase of the isotherm is equally informative. Notably, during the desorption phase, a hysteresis loop is observed as the gas pressure is decreased. This hysteresis is a critical indicator of the pore structure and surface energy of the material. In the case of T10/MoS_2_, the substantial desorption hysteresis suggests that some nitrogen molecules remain trapped within the mesoscale pores even as the pressure is reduced. This phenomenon is often associated with specific pore geometries, such as ink-bottle-shaped or slit-shaped pores commonly found in mesoporous materials. The nitrogen adsorption–desorption isotherms provide compelling evidence of the distinct pore structures of MoS_2_ and T10/MoS_2_. The distribution of pores in the material by BJH method is given in Fig. 5b which also shows the average pore size distribution in the range of 2–3 nm.

### Electrochemical characterization

**Figure 6:**
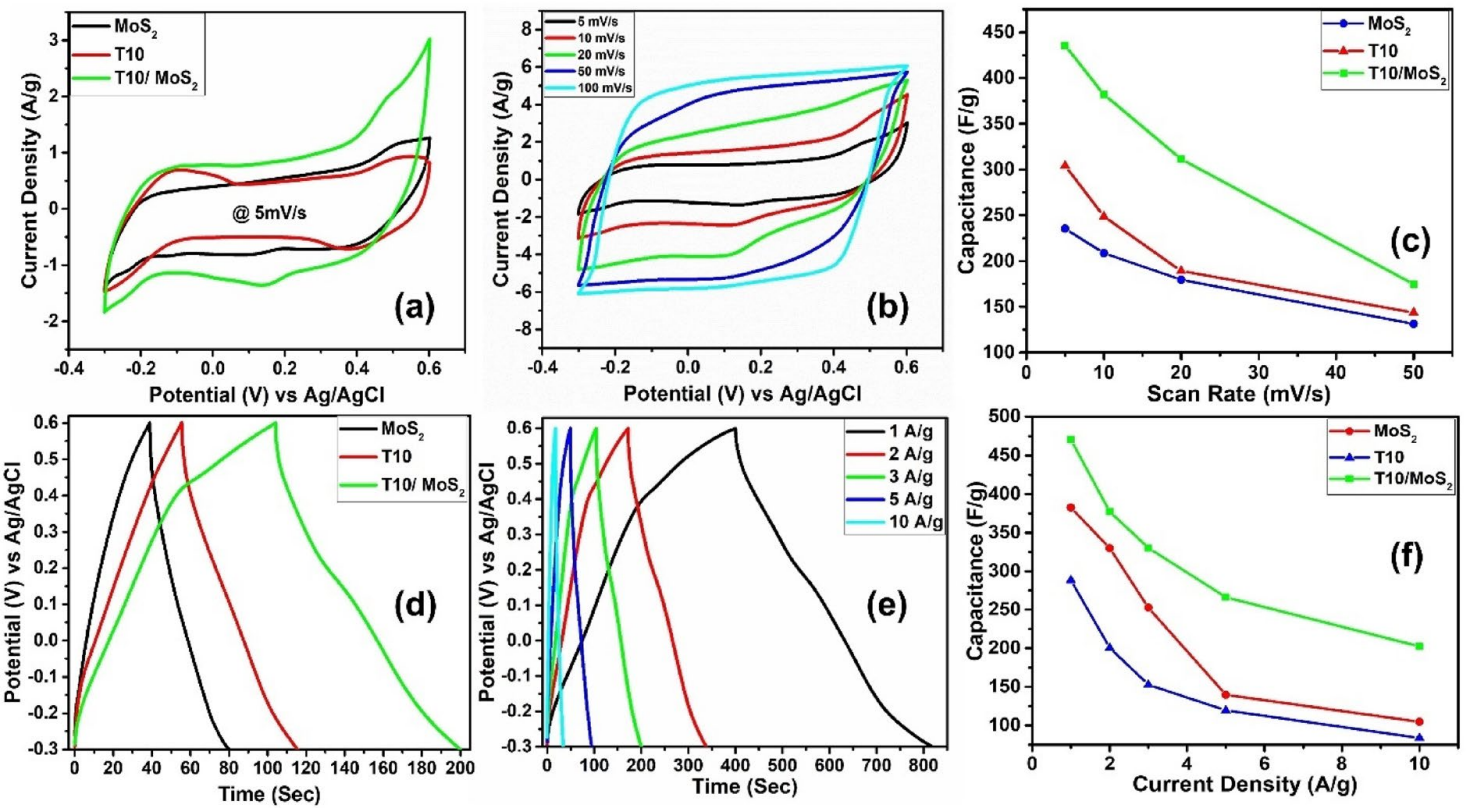
(**a**) CV comparison of MoS_2_, T10, and T10/MoS_2_ at 5 mV/s, (**b**) CV of T10/MoS_2_ at different scan rates, (**c**) Capacitance variation with scan rate, (**d**) GCD comparison of MoS_2_, T10, and T10/MoS_2_ at a current density of 3 A/g, (**e**) GCD of T10/MoS_2_ at different charge/discharge rates, and (**f**) Capacitance variation with current density.

After performing the material characterization, we assess the electrochemical activity of MoS_2_, T10, and T10/MoS_2_ electrodes in a 6 M KOH electrolyte. Figure 6a showed comparative cyclic voltammetry (CV) measurements of the various samples (MoS_2_, T10, and T10/MoS_2_) that were performed at a fixed scan rate of 5 mV/s. The safe potential range for each sample was between -0.3 and 0.6 V versus Ag/AgCl reference electrode. The sample T10 showed the redox activity at lower potential whereas the MoS_2_ sample showed higher electrochemical activity at higher potential. The synergistic combination of T10 and MoS_2_ can be observed in the T10/MoS_2_ sample which showed high electrochemical activity at lower and higher potential in CV. Further, the sample T10/MoS_2_ attained the highest CV area indicating the higher capacitance than MoS_2_ and T10. Upon calculating the capacitance from CV area using Eq. (S1), the sample T10/MoS_2_ delivered 436 F/g of specific capacitance as compared to pristine MoS_2_ (235 F/g) and T10 (304 F/g) electrodes at the scan rate of 5 mV/s. Further, CV characteristics of T10/MoS_2_ composite electrode is analyzed at different scan rates (1–100 mV/s) (Fig. 6b). CV curves at high scan rates are of rectangular shape which is attributed to the fast surface reaction due to the presence of densely packed redox active material at the surface. However, at the slow scan rate, obvious redox peaks can be observed which clearly mentioned the large number of redox reactions in bulk material that is required to attain high energy density. The present T10/MoS_2_ composite electrode-based supercapacitor thereby combines EDLC and Faradaic charge storage methods, boasting characteristics of both high energy and high-power densities. C_s_ values have been calculated from CV plots at various scan rates by using Eq. (S1). When calculated, T10/MoS_2_ delivered the C_s_ of 436 F/g at the scan rate of 5 mV/s. However, with increasing scan rate, the C_s_ of the electrodes decreases as shown in Fig. 6c. This effect might be due to the fact that the electrolyte ions may not have complete accessibility to the reactions sites in the bulk of the electrode material when the system is operated at high scan rates. The electrode retained 102 F/g of specific capacitance when scanned at the rate of 100 mV/s.

After CV analysis, we performed the GCD to observe the charge discharge characteristics and the rate performance of the electrodes (shown in Fig. 6d,e,f). All the discharge curve in Fig. 6d showed typical linear behavior with a negligible voltage drop indicating a low internal resistance^47^. The charge discharge characteristics of the electrodes indicates the fast surface and redox reactions characteristic of the capacitor type behavior. However, at slow discharge rates, a small non linearity can be observed which is due to the redox reactions in the system owing to the co-presence of TiO_2_, MoS_2_, and the redox active functional groups. Upon calculating the specific capacitance from the discharge curve, the T10/MoS_2_ sample delivered 320 F/g of capacitance at the current density of 3 A/g. Compared to this, the sample MoS_2_ delivered 152 F/g and T10 delivered 250 F/g of capacitance when discharged at the rate of 3 A/g. The lower capacitance value of MoS_2_ and T10 can be attributed to the absence of conductive channels in case of bulk MoS_2_ as well as the low SSA, whereas for T10 it can be attributed to the absence of large number of redox active groups at the surface. Further, to check the rate performance of the T10/MoS_2_, charging-discharging test has been performed at different current density as shown in Fig. 6e. All of the curves are showing typical linear discharge behavior. Figure 6f shows the variation of the C_s_ as a function of current density. Similar to the trend in CV rate performance, the GCD rate performance showed the decrease in C_s_ with increase in discharge rate. At high current densities, the bulk material might not support complete adsorption–desorption of electrolyte ions as well as the redox reactions which lead to the loss of capacitance with increased rate^48^. The sample T10/MoS_2_ showed 470 F/g of capacitance at the current density of 1 A/g. However, with the increase of tenfold current density i.e. 10 A/g, the material was capable of delivering a 202 F/g of specific capacitance. The equations used for the computation of C_s_ values derived from GCD data are provided in SI (Eq. (S2)).

The T10/MoS_2_ material showed redox peaks at lower scan rate and rectangular CV curves at higher scan rate. This is because with the change in scan rate, the charge storage behavior also got affected which is generally of two types: surface controlled and diffusion controlled. We used the CV curves of all three material to quantitatively analyze the surface and diffusion-controlled behavior. The current of the CV can be expressed as Eq. (1) in which current is directly proportional to the scan rate with some power constant b. The Eq. (1) can further be resolved into Eq. (2) which can be plotted to obtain the b values^41^.

1 $$i_{p} = av^{b}$$

2 $$log\left( {i_{p} } \right) = b log\left( v \right) + log a$$

**Figure 7:**
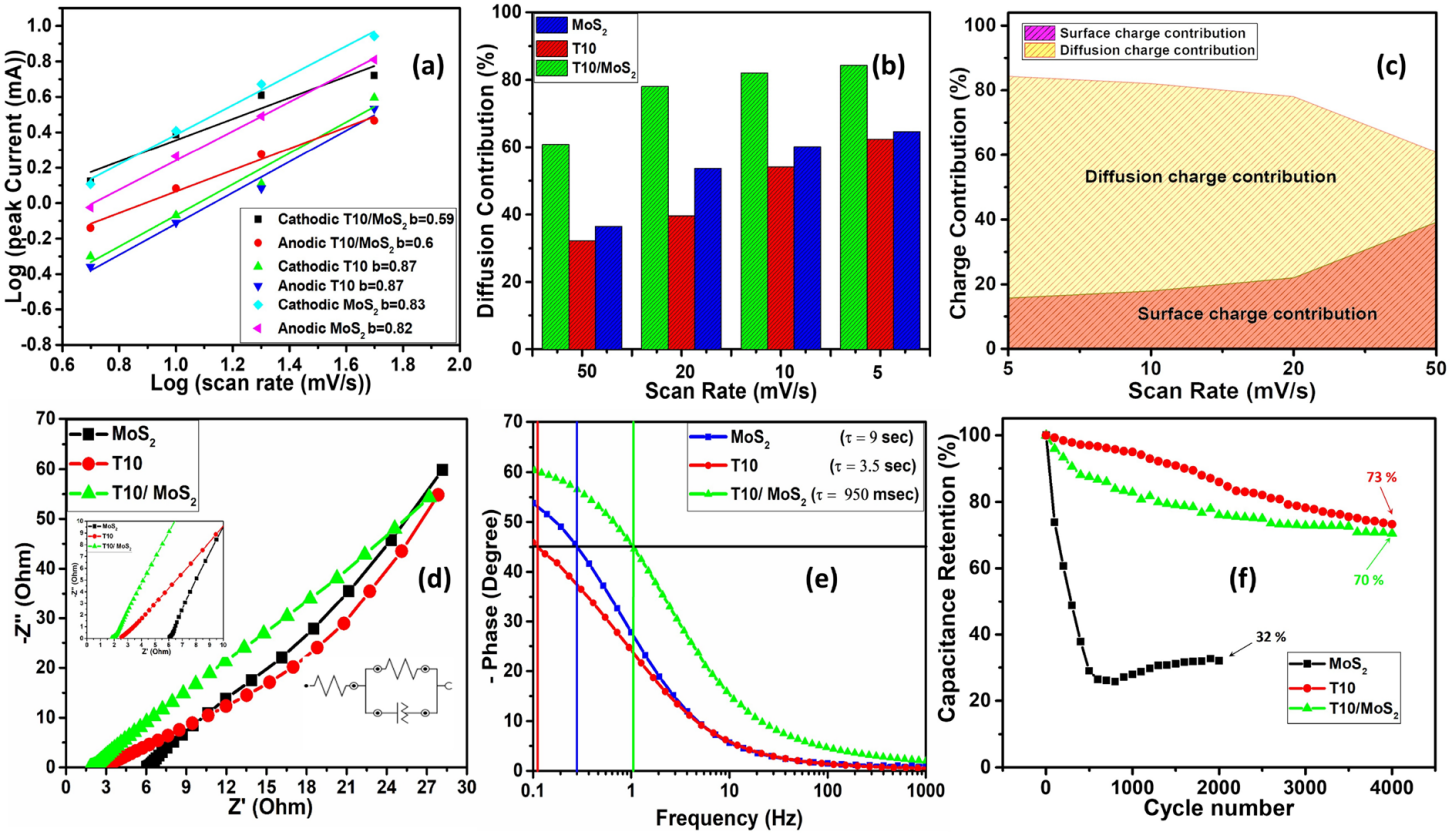
(**a**) b values for different samples, (**b**) diffusion current contribution, (**c**) surface and diffusion charge contribution for T10/MoS_2_, (**d**) Nyquist plot (inset: equivalent circuit), (**e**) Bode-Phase and relaxation time, and (**f**) cyclic stability test.

From the CV data of different scan rate, we plotted Fig. 7a for different materials using Eq. (2), we have determined the b values from the slope. b value is the indicator of capacitive or diffusion-controlled reactions. In detail, for capacitive controlled reactions, current in the CV increases directly propositional to the scan rate i.e., b can be taken as 1 in Eq. (1). On the other hand, for diffusion-controlled reactions, the current is directly proportional to the square root of scan rate i.e., b = 0.5 in Eq. (1). In contrast, b = 1 would point toward ideal surface-controlled process involving adsorption desorption of electrolyte and surface faradic reactions whereas b = 0.5 indicate the diffusion reactions in the bulk of material^42^. Therefore, b value in between the 0.5 to 1 indicate the mixture of diffusion and surface-controlled reactions. For T10/MoS_2_, the b value for anodic and cathodic current is equal to 0.6 and 0.59 respectively which is lower when compared to MoS_2_ (0.83 for cathodic and 0.82 for anodic) and T10 (0.87 for cathodic and 0.87 for anodic). The lower b value of T10/MoS_2_ indicate the higher percentage of diffusion-controlled reaction in the material which is probably due to the porous core with dense redox active surface facilitating the diffusion of ions in the bulk nanostructures. Apart from this, the b value for T10 and MoS_2_ is more surface controlled. The higher surface-controlled contribution from T10 is due to the open porous structure of carbon which can accumulate the electrolyte in the pores. During charging and discharging, the electrolyte is adsorbing/desorbing at the inner surface of carbon. On the other hand, the high surface-controlled contribution from MoS_2_ is due to inability of electrolyte to diffusion inside the bulk of MoS_2_ due to low porosity.

Apart from this, to calculate the surface and diffusion current distribution we have used Dunn et al. method with which surface ${I}_{s}$ and diffusion current ${I}_{D}$ can be related to the scan rate (ν) with the following Eq. ^41^

3 $${\text{Surface}}\;{\text{ controlled }}\;{\text{current}}:\;I_{s} = K_{1} \nu$$

4 $${\text{Diffusion}}\;{\text{ controlled }}\;{\text{current}}:\;I_{D} = K_{2} \nu^{{{\raise0.7ex\hbox{$1$} \!\mathord{\left/ {\vphantom {1 2}}\right.\kern-0pt} \!\lower0.7ex\hbox{$2$}}}}$$

5 $${\text{Total }}\;{\text{current:}}\;I = I_{s} + I_{D}$$

${K}_{1}$ and ${K}_{2}$ are the proportionality constant. Using Eq. (3), (4) and (5), we can write the Eq. (6) as follows

6 $$Or,\;\frac{I}{{\nu^{{{\raise0.7ex\hbox{$1$} \!\mathord{\left/ {\vphantom {1 2}}\right.\kern-0pt} \!\lower0.7ex\hbox{$2$}}}} }} = K_{1} \nu^{{{\raise0.7ex\hbox{$1$} \!\mathord{\left/ {\vphantom {1 2}}\right.\kern-0pt} \!\lower0.7ex\hbox{$2$}}}} + K_{2}$$

The equation shows the straight-line equation whose slope and intercept can deliver the value of K_1_ and K_2_ which further can be used to calculate the surface and diffusion current using Eq. (3) and (4). The calculated diffusion contribution for all the samples at different scan rate is given in Fig. 7b. T10/MoS_2_ showed 60% of current contribution from diffusion-controlled reaction which increased to 82% with the decrease of scan rate to 5 mV/s. At lower scan rate, ions have more time to diffuse in the bulk material and so lead to higher diffusion contribution but with increase in scan rate the bulk material contribution decreases due to insufficient time for ions to diffuse. Compared to the T10/MoS_2_, pristine MoS_2_ showed lower diffusion contribution (36% at 50 mV/s) which is due to inefficient porosity for the diffusion of ions in the bulk. On the other hand, the lower diffusion current of T10 (31% at 50 mV/s) can be attributed to the open porous structure and so contributing the higher surface contribution but exhibit low capacitance confirmed from GCD. T10/MoS_2_ exhibits this transition due to the combined effects of the porous core structure of T10 and the presence of redox-active MoS_2_ nanosheets on the surface. These factors collectively contribute to the observed shift from surface-controlled to diffusion-controlled current after recombination in the T10/MoS_2_ composite. This can be explained on the synergy between T10 and MoS_2_ in the composite which plays a crucial role. While T10's porous core enhances ion diffusion, MoS_2_ contributes redox-active surface sites. These redox-active sites promote fast surface-controlled reactions, but the porous core ensures that a significant portion of the ions is still involved in diffusion-controlled processes within the bulk of the composite. The diffusion and surface charge contribution at various scan rates for T10/MoS_2_ is also given in Fig. 7c corroborating the increase of the fraction of diffusion contribution with decrease in scan rate.

Further, the efficiency of the T10/MoS_2_ electrode has been confirmed by carrying out EIS analysis. The Nyquist plot showed in Fig. 7d indicate the small semicircle in high frequency region (starting) with a straight line in the low frequency region (tail). Nyquist plot indicates that both T10 and T10/MoS_2_ samples exhibit lower resistance, likely attributed to the reduced resistance resulting from the interconnected carbon network. Additionally, the semicircle in the Nyquist plot is not prominent. The less prominent semicircular nature of the Nyquist plot for the T10/MoS_2_ material can be attributed to the efficient and rapid charge transfer at the electrode–electrolyte interface. This phenomenon is a result of the unique properties of the T10/MoS_2_ composite, including the presence of redox-active MoS_2_ at the surface and the interconnected porous core network of TiO_2_/carbon. MoS_2_ facilitates fast surface redox reactions, while the porous core structure allows for efficient ion transport and charge distribution within the material. These factors work synergistically to minimize impedance associated with diffusional limitations, resulting in a Nyquist plot dominated by efficient charge transfer processes and a less pronounced semicircular feature. Although in MoS_2_, we used carbon black as conductive additive, but the interconnected carbon structure in T10 and T10/MoS_2_ helped minimize the interfacial charge transfer resistance at the electrode/electrolyte interface which is crucial factor for the transport of electrons. Further from the bode plot of frequency phase relationship, we have calculated the relaxation time for different material which correspond to the time taken by the supercapacitor to relax or discharge from a perturbed state as shown in Fig. 7e. It is a measure of the energy dissipation and is related to the internal resistance of the supercapacitor. A higher relaxation time implies a higher internal resistance, which can limit the supercapacitor's ability to deliver power. For the T10/MoS_2_ composite, the relaxation time has been calculated to 0.95 s which is much lower as compared to T10 (3.5 s) and MoS_2_ (9 s).

Both CV and GCD investigations revealed the enhanced C_s_ values of T10/MoS_2_ electrode when using 6 M KOH as an aqueous electrolyte. The excellent performance of T10/MoS_2_ can be attributed to the presence of large numbers of dense reaction sites at the surface allowing high energy density with porous core to support bulk material utilization. The cyclic stability of the T10/MoS_2_ electrode has also been investigated for all the electrodes (Fig. 7f). The T10/MoS_2_ electrodes retained 70% of its original specific capacitance even after 4000 charge–discharge cycles, clearly indicating the higher stability than the bulk MoS_2_ (32% capacitance retention). In comparison to T10, which maintained 73% of its capacitance retention, the performance of T10/MoS_2_ is lower. This is likely because MoS_2_, which is slightly soluble in the electrolyte, is present in T10/MoS_2_. MoS_2_ is also prone to thermodynamic/kinetic instability when oxidized, resulting in nanosheet degradation and the release of soluble molybdenum and sulfur species. This generates protons that can cause the remaining sheets to become colloidally unstable^42^. However, since in case of T10/MoS_2_, MoS_2_ is covalently bonded with T10 and hence showed lower degradation issue compared to bulk MoS_2_ whose capacitance decreases to 32% due to higher dissolution. Therefore, the main stability in the T10/MoS_2_ can be attributed to the presence of covalent bonds with the T10 which can’t be easily dissolved in electrolyte like in MoS_2_. On the other hand, T10 retained higher capacitance retention compared to T10/MoS_2_ due to its higher stability with the electrolyte.

### Performance analysis of T10/MoS_2_//T10/MoS_2_ solid-state symmetrical supercapacitor

**Figure 8:**
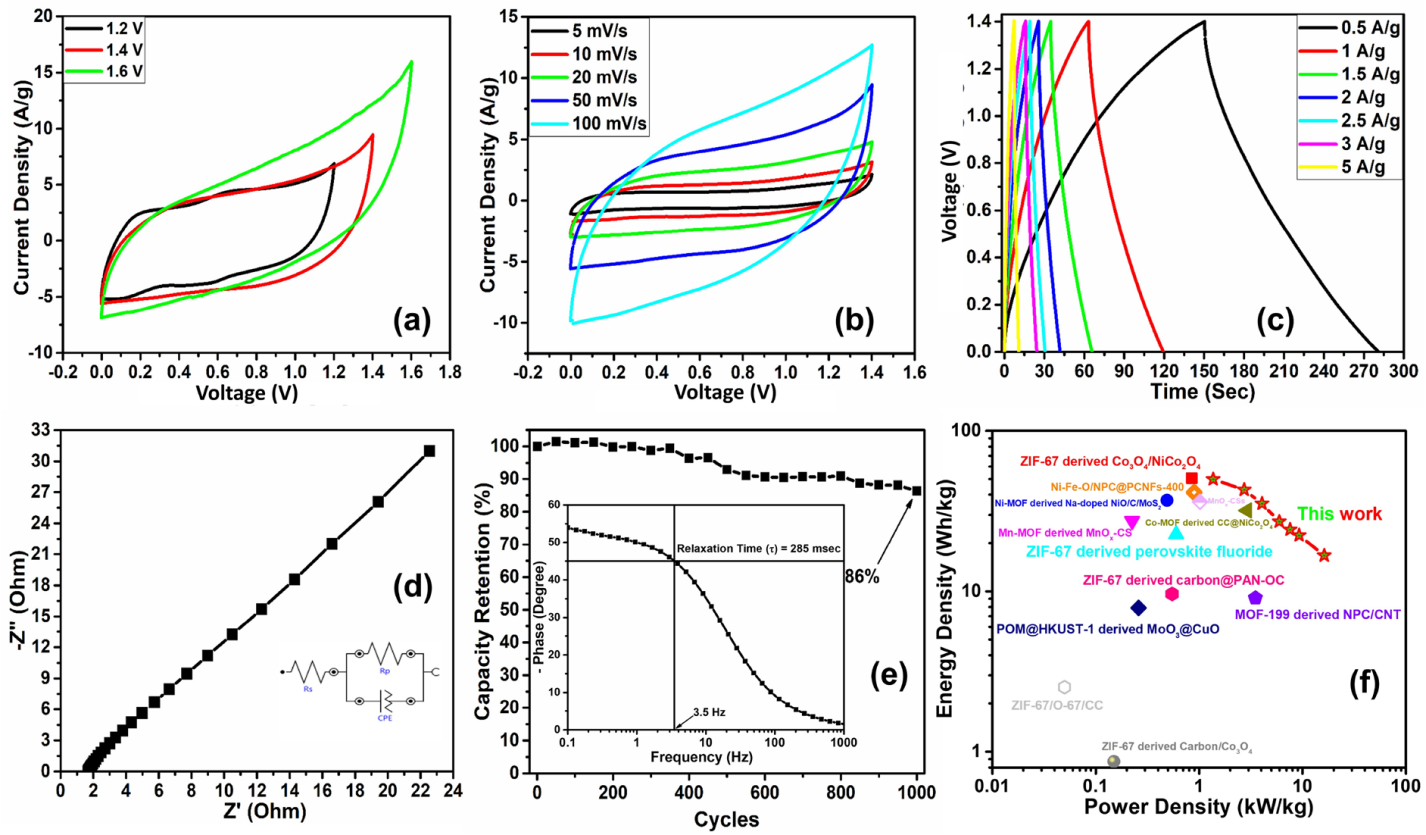
Electrochemical characterization of assembled symmetrical supercapacitor T10/MoS_2_//T10/MoS_2_ using 6 M KOH-PVA gel electrolyte. (**a**) Voltage window test, (**b**) CV at different scan rates, (**c**) GCD at different current densities, (**d**) Nyquist spectra (inset: equivalent circuit), (**e**) Cyclic stability test (inset: Bode phase plot), and (**f**) Ragone plot.

The results of T10/MoS_2_ in three electrode system showed outstanding electrochemical performance (with a 6 M KOH electrolyte). The studies were then expanded to evaluate the performance of a symmetrical supercapacitor made from two equal-weight T10/MoS_2_ electrodes taking 2 mg of mass loading on both electrodes and 6 M KOH-PVA gel electrolyte. The polymer gel electrolyte was sandwiched between the two electrodes where it also functioned as a separator to prevent the device from short circuiting. The assembled T10/MoS_2_//T10/MoS_2_ symmetrical supercapacitor was first operated under different potential window to find appropriate operating potential window. For this, CV curves were collected from 0 to 1.6 V (Fig. 8a). For the 0–1.6 V, the device showed abrupt increase in the positive current without significant changes in the negative current. Therefore, we have selected 0–1.4 V as a safe operating potential window for the device. CV behavior of the device at different scan rates are plotted in Fig. 8b. The CV patterns were typical for a supercapacitor device, possessing an almost perfect rectangular behavior. The calculation of C_s_ of the device has been performed using Eq. (S3). The device delivered 123.9 F/g of C_s_ at the scan rate of 5 mV/s which decreased to 67.5 F/g when the scan rate increased to 100 mV/s. The variation of C_s_ with scan rate is shown in Figure S5a.

Further we performed the GCD of the device in the potential window of 0–1.4 V as shown in Fig. 8c at different current densities ranging from 0.5 to 5 A/g. The device showed the linear charge–discharge characteristics which is a characteristic of a capacitor. When calculated using Eq. (S3), the device delivered a specific capacitance of 192 F/g at the current density of 0.5 A/g. Even the device retained the specific capacitance of 84.2 F/g, when the discharge current increased to 5 A/g. The dependence of C_s_ of the device upon current density is presented in Figure S5b. Further, to evaluate the charge transfer characteristics and resistance in the device, we performed EIS analysis. The Nyquist plot for the device is presented in Fig. 8d and bode plot (Phase vs. frequency) is given in the inset of Fig. 8e. The values of ESR of the device is estimated to 1.67 Ω whereas the relaxation time for the device has been calculated to 285 ms from the bode plot. Furthermore, to test the stability of the device, we performed the 1000 charging discharging cycles. The device retained 86% of the initial capacitance after 1000 charge–discharge cycles as shown in Fig. 8e. The energy density and the power density are the crucial parameters for a supercapacitor to determine its real potential as a device. Therefore energy (Es) and power (Ps) densities of the fabricated symmetric supercapacitor have been calculated (Eq. (S4) and (S5)). The associated Ragone plot is shown in Fig. 8f. The maximum attainable value of E_s_ was 49.2 Wh/kg at a P_s_ of 1.3 kW/kg. Even with the increase of power density to 16.25 kW/kg, the device retained 16.7 Wh/kg of energy density. As such, the excellent electrochemical performance of the herein proposed system can be attributed to two main synergistic factors, e.g., (i): the involvement of redox active surface (MoS_2_) and (ii) conducting, redox active, and porous core consist of TiO_2_/C network, which allowed the device to deliver high energy density by retaining its high-power density.

Table 1 summarizes a comparison between the current system and similar supercapacitor designs that have been previously reported. The T10/MoS_2_ electrode system has a lot of potential for creating supercapacitors that are incredibly efficient, according to the information that is currently available. Due to its straightforward and inventive production method, which enables the impregnation of metal oxide nanoparticles within the porous carbon matrix and the existence of densely packed redox sites on the MoS_2_ surface, T10/MoS_2_ exhibits better electrochemical performance. Moreover, covalent connection of MoS_2_ to the carbon framework adds to its stability by preventing it from dissolving in the electrolyte. Additionally, the electrode material can adapt to volume variations brought on by the introduction of electrolyte ions due to the flexible and porous carbon matrix.

**Table 1:** Comparison of different MOF based electrode materials for supercapacitor applications.

S. No	Electrode material	Electrolyte	Voltage window(V)	C_s_(Fg^−1^) (I_d_ (A/g))	${E}_{D}$(Wh/kg)/${P}_{D}$(kW/g)	References
1	ZIF-67/O-67/CC	3 M KOH	− 0.2 to 0.5	80 (1.5)	2.53/0.05	^43^
2	ZIF-67 derived Carbon/Co_3_O_4_	3 M KOH	− 0.2 to 0.5	51 (1.5)	0.87/0.15	^44^
3	ZIF-67 derived carbon@PAN-OC	1 M H_2_SO_4_	− 0.5 to 0.1	270 (1)	9.64/0.55	^45^
4	ZIF-67 derived perovskite fluoride	3 M KOH	0 to 0.6	368 (2)	22.7/0.6	^46^
5	Polyoxometalates@HKUST-1 derived MoO_3_@CuO	1 M LiOH	− 0.3 to 0.5	86.3 (1)	7.9/0.26	^47^
6	ZIF-67 derived Co_3_O_4_/NiCo_2_O_4_	6 M KOH	-0.1 to 0.6	126 (1)	50.6/0.85	^48^
7	Ni-Zn-BTC derived ZnO/NiO	3 M KOH	0 to 0.4	172 (0.5)	–	^49^
8	Yolk–shell Ni–Zn-BDC derived NiO/ZnO	3 M KOH	0 to 0.5	497 (1.3)	–	^50^
9	Fe^III^-MOF-5 derived star fish shaped Co_3_O_4_/ZnFe_2_O_4_	6 M KOH	− 0.9 to 0.6	326 (1)	–	^51^
10	Co-MOF derived CC@NiCo_2_O_4_	2 M KOH	0 to 0.6	483 (1)	31.9/2.9	^52^
11	Mn-MOF derived MnO_*x*_-CS	1MNa_2_SO_4_	0 to 1	220 (1)	27.5/0.225	^53^
12	MOF-199 derived NPC/CNT	6 M KOH	− 0.1 to − 1.1	194 (2)	9.1/3.5	^54^
13	Ni-MOF derived Na-doped NiO/C/MoS_2_	2 M KOH	0 to 0.5	1779 (0.6)	36.9/0.49	^55^
14	Ti-MOF derived TiO2/carbon/MoS_2_	6 M KOH	−0.3 to 0.6	470 (1)	49.2/1.3	This work

## Conclusions

The present work demonstrates the usefulness of a hybrid T10/MoS_2_ composite as an efficient supercapacitor electrode material. The material synthesized by the pyrolysis of MIL-125(Ti) at 1000 °C to obtain a micro/meso porous carbon structure with the impregnation of TiO_2_ nanoparticles. Further, the surface of the T10 material has been decorated with MoS_2_ nanosheets to improve the redox activity. The T10/MoS_2_ composite attained the surface redox activity with the porous core to have diffusion of ions so that bulk of the material can be utilized. T10/MoS_2_ electrode-based supercapacitor is characterized by excellent electrochemical performance which delivered 470 F/g of specific capacitance at the current density of 1A/g. Due to favorable distribution of meso and micropores, carbon fraction of the composite material contributed to the realization of a high-power density. On the other hand, the redox nature of the MoS_2_ and TiO_2_ fraction led to the surface and bulk redox reaction giving additional pseudocapacitance to attain the higher energy density. The excellent performance of the assembled device will make it useful in diverse fields of application in which high energy and high-power density specifications are demanded. The T10/MoS_2_ electrode can be developed suitably for a single symmetrical device or a series of devices.

### Supplementary Information

Supplementary Information.

## Data Availability

The datasets used and/or analyzed during the current study are included in the published article. The raw data used are available upon request from the corresponding author.
