# Supplementary material for: Diffusion controlled electrochemical analysis of MoS2 and MOF derived metal oxide–carbon hybrids for high performance supercapacitors
Source: Sci Rep. 2023 Nov 24;13:20675. doi: 10.1038/s41598-023-47730-4 (PMC10674017; doi:10.1038/s41598-023-47730-4)
Supplement: Supplementary file 1 — Supplementary Information. [file 41598_2023_47730_MOESM1_ESM.docx]

**Supporting Information**

**Diffusion controlled electrochemical analysis of MoS_2_ and MOF derived metal oxide-carbon hybrids for high performance supercapacitors**

Vishal Shrivastav^1$*^, Mansi^2$^, Prashant Dubey^3^, Vaishali Shrivastav^4^, Ashwinder Kaur^5^, Marcin Hołdyński^1^, Agnieszka Krawczyńska^6^, Umesh K. Tiwari^2^, Akash Deep^7^, Wojciech Nogala^1*^, and Shashank Sundriyal^8*^

^1^Institute of Physical Chemistry Polish Academy of Sciences, Kasprzaka 44/52, 01-224 Warsaw, Poland;

^2^CSIR-Central Scientific Instruments Organisation, Sector 30-C, Chandigarh, 160030, India;

^3^Advanced Carbon Products and Metrology Department, CSIR-National Physical Laboratory (CSIR-NPL), New Delhi 110012, India;

^4^Guru Nanak Dev University College, Chung, Punjab 143303, India;

^5^Department of Physics, Punjabi University, Patiala, 147002, India;

^6^Faculty of Materials Science and Engineering, Warsaw University of Technology, Wołoska 141 Str., 02-507 Warsaw, Poland;

^7^Institute of Nano Science and Technology (INST), Sector-81, Mohali, 140306 Punjab, India;

^8^Regional Center of Advanced Technologies and Materials, The Czech Advanced Technology and Research Institute (CATRIN), Palacký University Olomouc, Šlechtitelů 27, 779 00 Olomouc, Czech Republic;

**^$^Both authors contributed equally**

***Correspondence:** [sundriyal.s.1991@ieee.org](mailto:sundriyal.s.1991@ieee.org) (**Dr. Shashank Sundriyal**), [vshrivastav@ichf.edu.pl](mailto:vshrivastav@ichf.edu.pl) (**Dr. Vishal Shrivastav)**, [wnogala@ichf.edu.pl](mailto:wnogala@ichf.edu.pl) (Dr. Wojciech Nogala)

**S1. Experimental**

**S1.1. Materials**

Thiourea (CH_4_N_2_S), N-methyl-2-pyrrolidone (NMP), benzene-1,4-dicarboxylate (1,4-BDC), Sodium molybdate dihydrate (Na_2_MoO_4_·2H_2_O), titanium tetra-n-butoxide, polyvinyl alcohol (PVA), and Polyvinylidene fluoride (PVDF) were purchased from Merck. Dimethylformamide (DMF), sulphuric acid, and methanol were purchased from Merck. Ketjen black (EC-600JD) was purchased from Nanografi.

**S1.2. Synthesis of Ti-BDC MOF**

To synthesize Ti-BDC MOF, a solution of 2.4 ml of titanium tetra-n-butoxide, 2 g of 1,4-BDC, 36 ml of DMF, and 4 ml of methanol was stirred for 30 minutes. The resulting solution was then transferred to a Teflon-lined stainless steel autoclave and heated at 150°C for 24 hours in a hot air oven. The product obtained was a white yellowish precipitate, which was then centrifuged, washed three times with DMF and methanol, and dried in a vacuum oven at 80°C for 24 hours.

**S1.3. Synthesis of TiO_2_/C composite**

TiO_2_/Carbon structure has been produced by pyrolyzing Ti-MOF at different temperature under inert gas atmosphere. For this, a sample of Ti-MOF with a known quantity was put in an alumina boat and then heated in a tube furnace (Ar environment). The ramp rate for the pyrolysis temperature is kept at 5 ^o^C min^-1^, which is further maintained for 4 hours after reaching the pyrolysis temperature. The sample is cooled at the same ramp rate after 4 hours of pyrolysis. The Ti-MOF is pyrolyzed at three different temperatures (600, 800, and 1000 ^o^C), and given the designations Tn (where n is 6, 8, and 10, based on the pyrolysis temperature 600, 800, and 1000 ^o^C respectively, e.g. T10 for TiO_2_/carbon pyrolyzed at 1000 ^o^C).

**S1.4. Synthesis of TiO_2_/Carbon/MoS_2_ (T10/MoS_2_)**

To synthesize T10/MoS_2_, Na_2_MoO_4_·2H_2_O (3.629 g) and TiO_2_/Carbon (50 mg) were dissolved in 30 ml of DI water. Meanwhile, in another beaker, thio urea (3.4254 g) was dissolved in 30 ml of water. Both solutions were sonicated for 30 min and then mixed while stirring at room temperature for 30 min. The resulting mixture was transferred into a Teflon-lined stainless-steel autoclave and heated at 200°C for 12 h. The product was then washed with water and ethanol twice, and dried at 80°C for 10 h to obtain T10/MoS_2_. For MoS_2_ synthesis, the same steps were followed but without the addition of TiO_2_/Carbon.

**S1.5. Preparation of polymer gel electrolyte**

A polymer gel electrolyte consisting of 6M KOH-PVA was prepared. Firstly, 1.5 gm of PVA was dissolved in 10 mL of deionized water and heated while stirring at 95ºC until a clear gel was obtained. The gel was then allowed to cool to room temperature (25±2ºC), after which 10 mL of 12 M KOH were added to make a 20 mL gel solution.

**S1.6. Characterization**

Various instrumentation techniques such as SEM, XRD, Raman, and BET, were used for the characterization of material. Raman spectra were recorded on a Renishaw (Invia) system with a 532-nm laser source. Belsorp Max system (Microtrac) were used to perform N_2_ adsorption-desorption measurements. X-ray diffractometer (XRD) from Bruker D8 Advance, λ = 1.54 Å were used to record the XRD data of the samples. Scanning electron microscopy of the samples has been performed with FEI Nova NanoSEM 450. X-ray photoelectron spectroscopy (XPS) analysis were carried out using a Microlab 350 (Thermo Electron) spectrometer. TEM has been performed on JEOL 1200 at 200kV.

**S1.7.** **Electrochemical measurements**

The electrochemical measurements, such as cyclic voltammetry (CV), electrochemical impedance spectroscopy (EIS), and galvanostatic charge-discharge (GCD) were conducted using a PGSTAT 302N instrument from Autolab (Metrohm). A three-electrode cell containing reference (Ag/AgCl), counter (Pt), and working electrodes was utilized for all electrochemical measurements. To prepare the working electrode, a mixture of the active T10/MoS2 composite or T10 material, polyvinylidene fluoride (PVDF) binder, and a weight-to-weight ratio of 9:1 was used. A uniform slurry of this mixture was prepared in N-methyl pyrrolidone (NMP) and then coated over a graphite foil. For MoS_2_, the same method has been used with the addition of conductive carbon black and PVDF in the 8:1:1. The prepared electrodes were dried at 80 °C for 20 hours in a vacuum oven. The material density of T10/MoS_2_ composite over an electrode was estimated as 2 mg/cm^2^. Further, using T10/MoS_2_ composite as an electrode material, a symmetrical supercapacitor device was assembled. It consisted of two identical electrodes (1.5 cm × 1.5 cm) with equal masses of TiO_2_/C material over them (4 mg). PVA-6 M KOH was used as a polymer gel electrolyte in the device.

**Figure S1.** XRD of Ti-MOF.


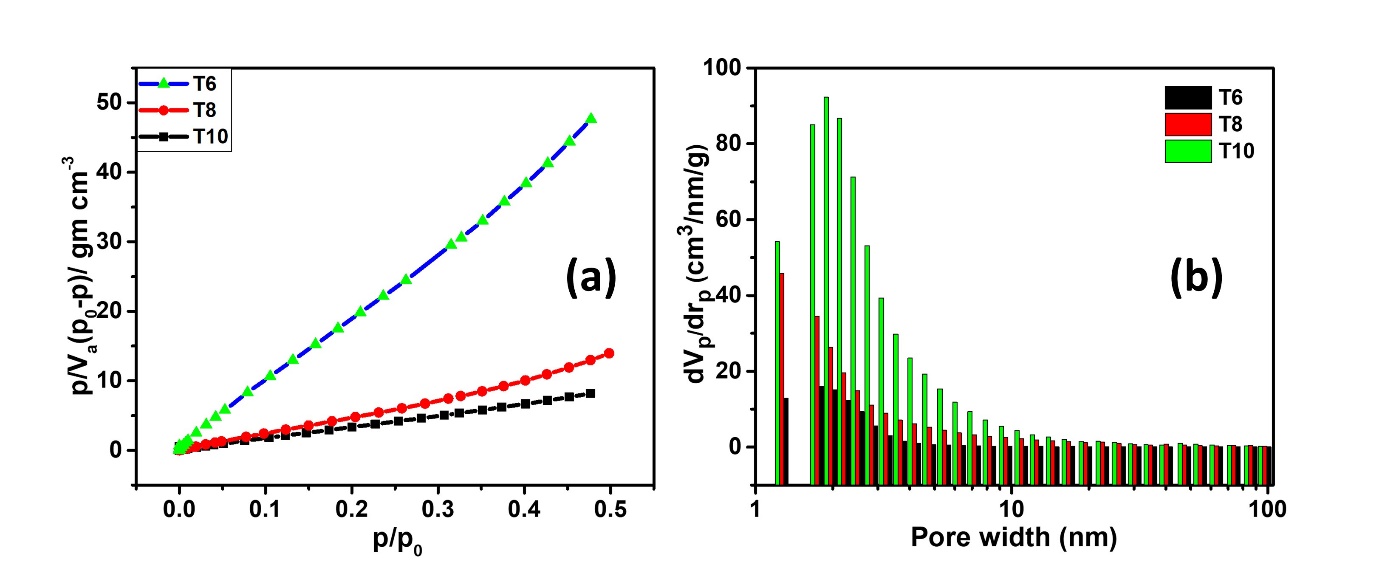


**Figure S2.** (a) BET Surface area test and (b) pore size distribution.


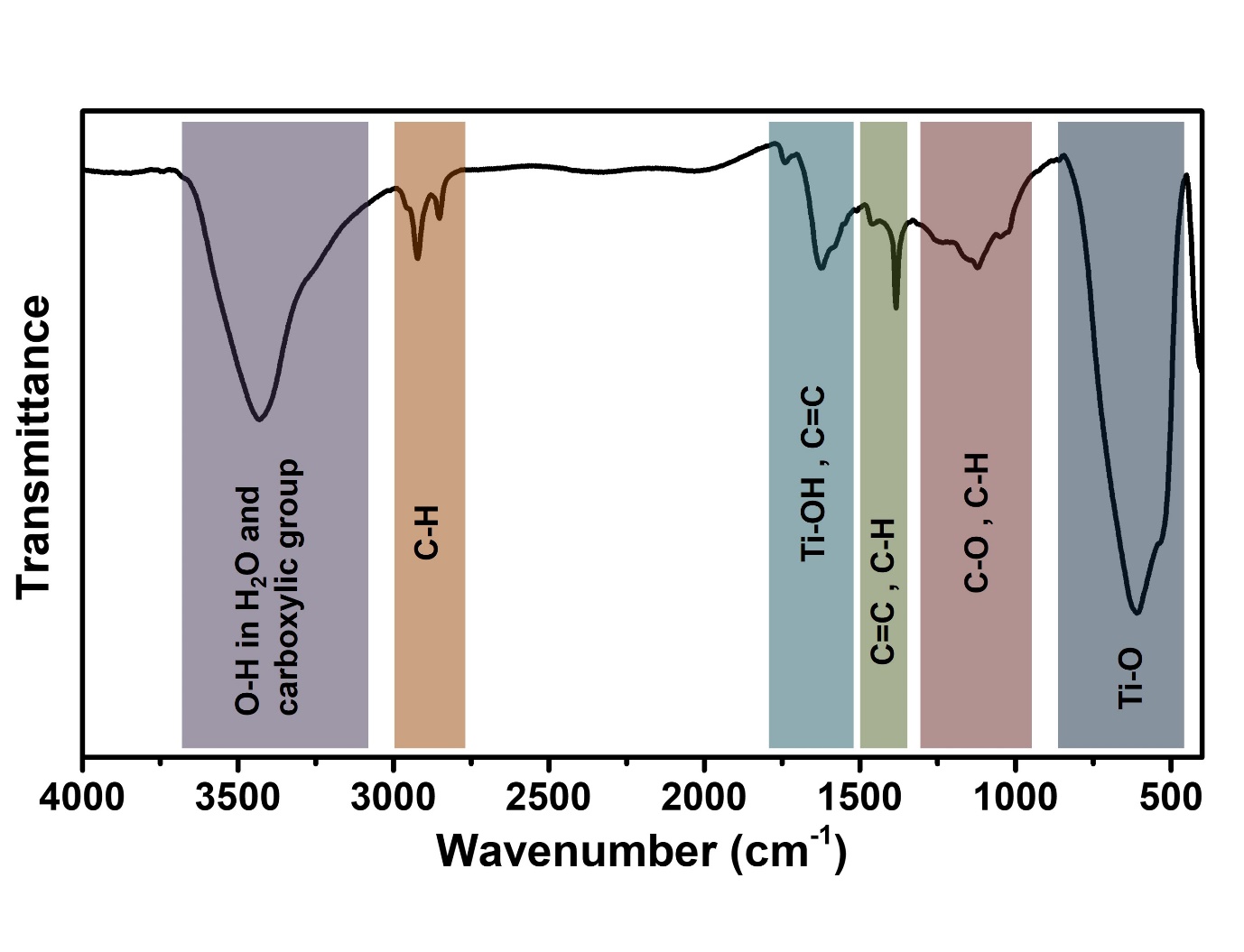


**Figure S3.** FTIR of T10 (TiO_2_/carbon).


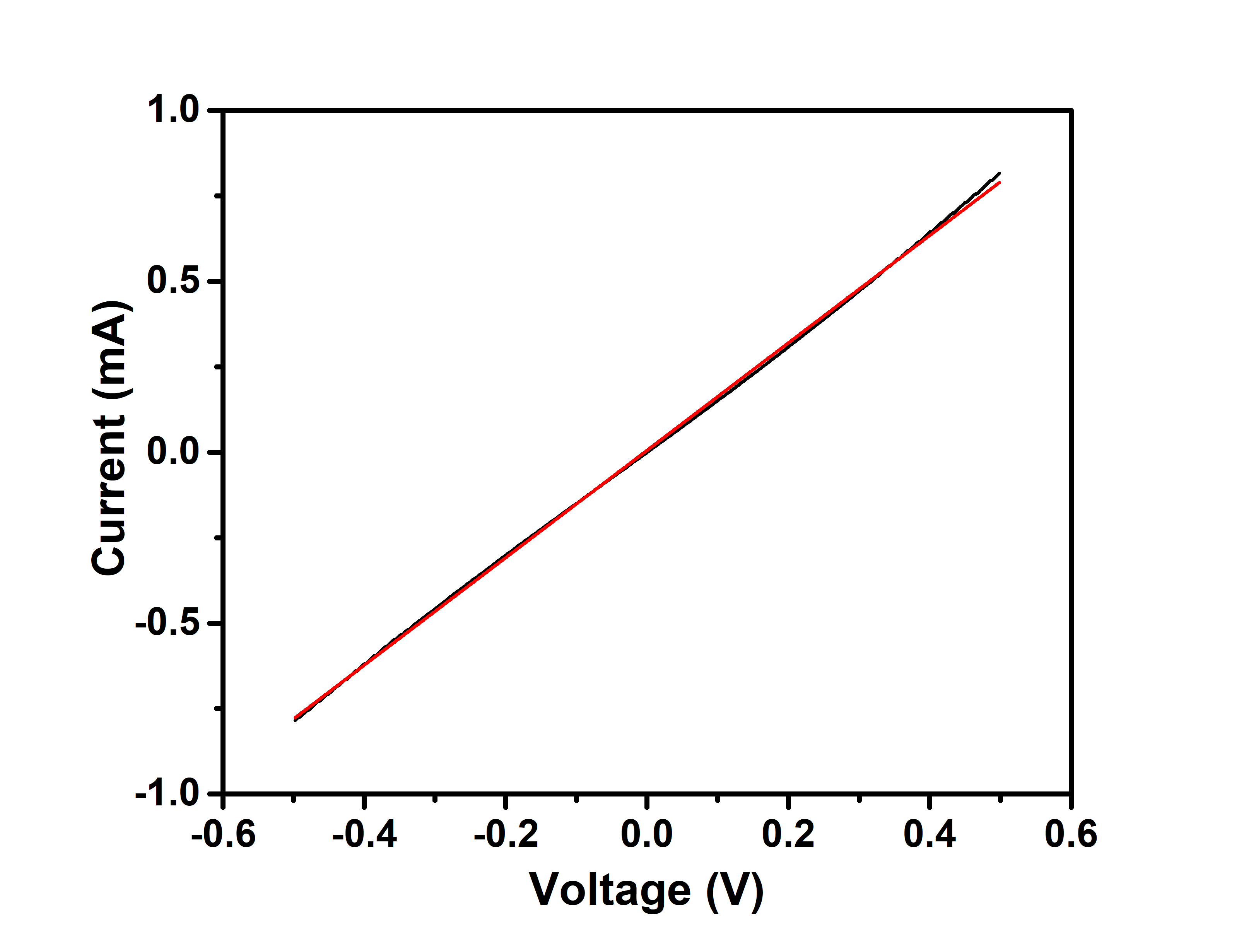


**Figure S4.** Current-Voltage (I-V) studies for T10.

**Calculations of electrochemical parameters**

1. **Using a three-electrode system**

Specific capacitance using cyclic voltammetry (CV) curves was calculated using the following equation:

$\text{C}_{\text{s}}=\frac{1}{\text{m}s\text{(}\Delta\text{V)}}\int_{\text{V}_{\text{a}}}^{\text{V}_{\text{c}}} \text{I(V)dV}$ (Eq. S1)

where (∫*IdV*) = integral area of CV curve, m = mass of active material on the electrode in g, ΔV = total potential deviation of the voltage window, and s = scan rate in mV s^-1^.

The galvanostatic charge–discharge (GCD) plots were used to calculate various parameters as mentioned under curves calculations:

1. Specific capacitance:

$\text{C}_{\text{s}}=\frac{\text{I}\times\Delta\text{t}}{\text{ΔV}\times\text{m}}$ (Eq. S2)

where I = discharge current, m = mass of active materials on the electrode in g, Δt = discharge time and ΔV = total potential deviation of the voltage window.

1. **Using symmetrical supercapacitor (two-electrode system)**

GCD based studies were used to calculate various parameters as elaborated by the following equations:

$\text{C}_{\text{s}}=\frac{4\times\text{I}\times\Delta\text{t}}{\text{ΔV}\times\text{m}}$ (Eq. S3)

where I = discharge current, m = sum of the masses of active materials on both the electrodes in g (m^+^= m^-^ for symmetrical supercapacitor), Δt = discharge time and ΔV = total potential deviation of the voltage window.

$\text{Es}=\frac{1\times\text{C}_{\text{s}}\times\text{(}\Delta\text{V}^{2}\text{)}}{4\times2\times3.6}$ (Eq. S4)

$\text{Ps}=\frac{\text{Es}\times3600}{\Delta\text{t}}$ (Eq. S5)

where C_s_ = specific capacitance, Δt = discharging time, ΔV = total potential deviation of the voltage window


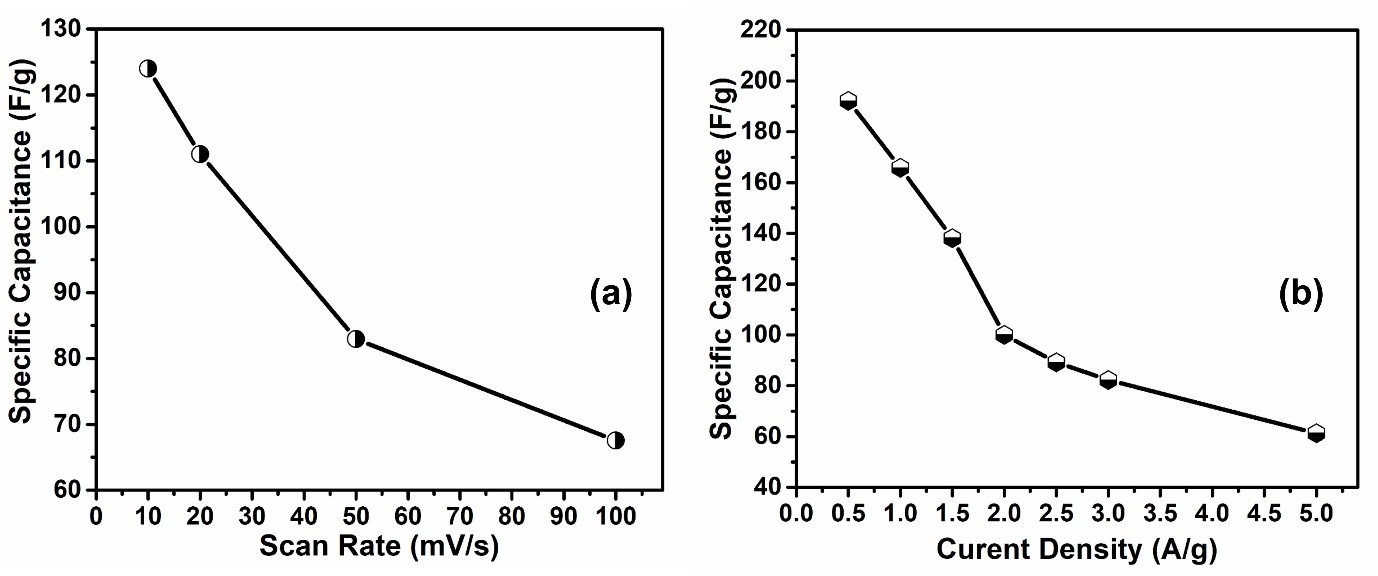


**Figure S5.** Rate performance of device: (a) specific capacitance vs scan rate and (b) specific capacitance vs current density.
